# Optimal Motion Planning in GPS-Denied Environments Using Nonlinear Model Predictive Horizon

**DOI:** 10.3390/s21165547

**Published:** 2021-08-18

**Authors:** Younes Al Younes, Martin Barczyk

**Affiliations:** Department of Mechanical Engineering, University of Alberta, Edmonton, AB T6G 1H9, Canada; alyounes@ualberta.ca

**Keywords:** motion planner, path planning, nonlinear model predictive approach, feedback linearization, dynamic obstacle avoidance, drone vehicle

## Abstract

Navigating robotic systems autonomously through unknown, dynamic and GPS-denied environments is a challenging task. One requirement of this is a path planner which provides safe trajectories in real-world conditions such as nonlinear vehicle dynamics, real-time computation requirements, complex 3D environments, and moving obstacles. This paper presents a methodological motion planning approach which integrates a novel local path planning approach with a graph-based planner to enable an autonomous vehicle (here a drone) to navigate through GPS-denied subterranean environments. The local path planning approach is based on a recently proposed method by the authors called Nonlinear Model Predictive Horizon (NMPH). The NMPH formulation employs a copy of the plant dynamics model (here a nonlinear system model of the drone) plus a feedback linearization control law to generate feasible, optimal, smooth and collision-free paths while respecting the dynamics of the vehicle, supporting dynamic obstacles and operating in real time. This design is augmented with computationally efficient algorithms for global path planning and dynamic obstacle mapping and avoidance. The overall design is tested in several simulations and a preliminary real flight test in unexplored GPS-denied environments to demonstrate its capabilities and evaluate its performance.

## 1. Introduction

Throughout the last century, injuries and fatalities in subterranean environments have remained a major concern around the world. For example, mine workers are vulnerable to hazards such as cave-ins, underground flooding, and gas explosions. Unmanned vehicles can play a key role in performing both tedious and dangerous tasks, for instance air quality sampling, tunnel inspections, and search-and-rescue missions. A flying drone is a particularly attractive platform for underground operations due to its abilities to move quickly, traverse any terrain, navigate through tight spaces, and capture data from any angle. Recent advances in robotics have motivated research into designing novel path planning approaches, allowing the vehicle to plan safe paths and navigate through previously unknown environments.

Path planning is a computational problem to generate and follow a collision-free trajectory from one point to another [1]. It has many applications, such as robotic surgery [2], driverless cars [3], automation [4], and mining [5]. An extensive amount of research has been conducted in the field of path planning for autonomous vehicles [3,6]. However, most of the presented approaches provide non- or sub-optimal solutions and do not account for the dynamics of the vehicle, instead treating it as a kinematic model with velocity inputs [1], for instance a unicycle or kinematic car [7]. Moreover, navigating through dynamic and unknown environments is a challenging task as it requires safe navigation around both static and dynamic obstacles, which adds computational load for the onboard computer of the autonomous vehicle. Nonlinear Model Predictive Control (NMPC [8]) is an attractive methodology to address the above-named challenges, since it is capable of predicting optimal trajectories, accounts for the dynamics of the plant, and supports hard state constraints which can be used to model either static or dynamic obstacles.

**Figure 1:**
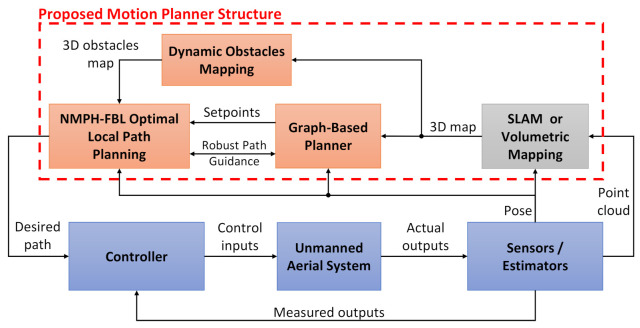
Block diagram of the proposed global motion planner.

The goals of this paper are twofold. The first goal is presenting the design of a local path planning approach and applying it to a multi-rotor drone. The nonlinear dynamics of a drone makes it an excellent test candidate for this work. The path planning approach is based on recent work by the authors [9]. It combines a variant of NMPC, named Nonlinear Model Predictive Horizon (NMPH), which employs the Feedback Linearization (FBL) technique [10,11] to reduce the non-convexity of the optimization problem and thus provide faster solutions for the path planning problem. NMPH provides feasible solutions, generates smooth and collision-free paths, supports moving obstacles, is able to run in real-time, and reduces battery draw by minimizing abrupt drone motions. The second goal is developing a global motion planner for the drone to explore a subterranean environment. This operates by building a map of the environment and guiding the vehicle to unexplored areas within this map using a graph-based planner. The global motion planner is a design that integrates the local path planner design from the first goal with a graph-based planner named GBPlanner [5]. We propose a choice of computationally efficient algorithms for obstacle mapping and avoidance, plus robust path guidance. A block diagram of the proposed global motion planner design is shown in Figure 1.

The contributions of this work are as follows:

- A recently proposed trajectory generation algorithm (NMPH) is used for local path planning of the drone. The NMPH is integrated inside the global motion planner and produces optimal local trajectories for the drone vehicle in real-time.
- A methodological two-layer global motion planner design is proposed. The first layer utilizes a graph-based planner to generate terminal setpoints for the second layer, which uses the NMPH design to generate continuous optimal paths from the vehicle’s current pose to the terminal setpoint in real time.
- Efficient algorithms for obstacle mapping and avoidance are proposed which produce models of static and dynamic obstacles used by the NMPH to generate safe and collision-free paths in a dynamically changing environment.
- A robust path guidance algorithm is implemented to avoid the risk of NMPH getting trapped into a local minima.
- The overall design is implemented using quadcopter and hexacopter drone dynamics, enabling navigation through unknown, dynamic and GPS-denied environments.
- Several simulation results and a preliminary experiment are presented in this work to validate the proposed approach.

The remainder of this paper is organised as follows. Section 2 surveys literature related to our work. Section 3 presents the problem formulation of NMPH and its integration with feedback linearization. The two-layer global motion planner design and choices of algorithms to provide robust path planning and obstacle avoidance are discussed in Section 4. System dynamic models of and implementation of our design on rotary-wing drones are presented in Section 5. In Section 6, various simulation and experimental results are presented to evaluate and validate the proposed approach. Finally, concluding remarks are given in Section 7.

## 2. Related Work

Path planning for an autonomous robot in an unknown, GPS-denied and dynamically changing environment is a challenging task, since the robot needs to plan trajectories that consider the vehicle’s relative motion with respect to the surrounding obstacles. The path planning problem itself has been thoroughly studied in the literature and can be classified into three main categories: search-based, sampling-based, and optimization-based methods.

The search-based methods, a.k.a. grid-based, discretize the environment map into a graph of grids and use a search algorithm to find a collision-free path through these grids [6]. The two fundamental graph search algorithms are Breadth-First Search (BFS) and Depth-First Search (DFS) [12]. BFS is based on a first-in-first-out queue and can produce an optimal solution if the graph is uniformly weighted. Meanwhile, a last-in-first-out stack is used in DFS until the goal is reached, but no optimality is guaranteed.

One of the most widely used optimal searching algorithms for quickly finding the shortest path is the Dijkstra algorithm [13]. It directs the search towards unvisited nodes, then calculates and updates the shortest distances to the neighbor nodes from the root node. It keeps doing this until all the nodes are visited. Meanwhile, A* [14] is a commonly used algorithm for path planning. A* is an extension to Dijkstra algorithm, where it combines the cost search with heuristics that guide the search towards the goal point to achieve quicker searching performance. Many extensions of A* have been proposed, for instance Lifelong Planning A* (LPA*) [15] was developed to support changes in the environment without recalculating the entire graph, D* Lite [16] extends LPA* to re-plan the path while the robot is moving, Anytime Repairing A* (ARA*) [17] improves the optimality of the path by reusing suboptimal solutions from previous executions, and Hybrid-state A* [18] generates the graph based on the robot dynamics and thus searches for a dynamically feasible path.

The sampling-based methods are considered one of the main motion planning methods for robots with a high number of Degrees-Of-Freedom (DOF) [19]. In these methods, feasible robot poses are randomly sampled to form admissible paths. Probabilistic Road-Map (PRM) [20] and Rapidly-Exploring Random Tree (RRT) [21] are the fundamental sampling-based methods for motion planning. In PRM, a graph is built from random configurations and connected using a local planner (for instance Dijkstra’s searching algorithm for the shortest path between two configuration). PRM is complete but does not necessarily provide an optimal path solution. The RRT method is designed to randomly build a space-filling tree of vertices and edges inside a complex environment to find a feasible path to the goal node. However, the RRT-generated paths are not optimal [22]. Asymptotic optimality of paths can be achieved by employing various extensions of RRT, such as RRT* and Rapidly-Exploring Random Graph (RRG) methods [22]. RRG constructs a graph by connecting new samples with all nodes within a specified distance, then finding the shortest path using a local planner such as the Dijkstra algorithm. Meanwhile, RRT* searches the local nodes and finds the shortest path from the start to end nodes.

In general, there are three main limitations of the search- and sampling-based motion planning approaches. First, they do not account for the constraints imposed by the robot dynamics, even if some support kinematic and/or dynamic constraints (e.g., velocity and/or acceleration limits, respectively) [23]. A second limitation is consistency, since for several executions the algorithms may not produce identical trajectories between a start and goal configuration in the very same environment. Third, the computational load of these methods generally prevents them from being able to actively regenerate paths while moving between the start and goal configuration, which makes motion planning a difficult task in dynamic environments. However, some optimization-based methods can overcome these limitations, and the present work is directed at using optimization for planning safe, consistent, and time-efficient paths which also respect the dynamics of the vehicle. This last feature allows generating smooth trajectories for the robot vehicle, avoding the jerky motions and rapidly changing trajectories often generated by other planning methods [1].

The optimization-based approaches solve a constrained non-convex optimization problem to smooth the trajectory generated by other methods. Some optimization-based methods use cost-gradient information of a trajectory’s waypoints for refinement purposes, for instance CHOMP [24], Trajopt [25], and STOMP [26]. Other optimization-based methods are more closely related to optimal control, which focuses on system dynamics more than collision prevention. Examples include dynamic programming [27], LQR-based [28], and Model Predictive Control (MPC) [29].

One of the challenges of using an optimization-based path planning approach is accounting for obstacle constraints at each time instant the optimization problem is solved, especially for real-time implementations [30]. For a small number of obstacles, it has been demonstrated that finding local optimal trajectories is possible with MPC in outdoor environments [31]. Conversely, increasing the number of obstacles and considering 3D and dynamic environments makes the optimization problem much more computationally expensive to find feasible paths in real-time.

Our proposed formulation addresses the above challenges in two ways. First, it reduces the non-convexity of the optimization problem by combining the nonlinear plant model with a feedback linearization. Second, it maps obstacles as possibly moving volumes, and adaptively introduces state constraints modeling them in order to efficiently find local solutions. Our proposed design is integrated with a graph-based exploration planner [5] in order to provide global motion planning capabilities.

## 3. Nonlinear Model Predictive Horizon for Path Planning

The Nonlinear Model Predictive Horizon is a recently proposed methodology by the authors [9]. The purpose of NMPH is to generate optimal reference trajectories for closed-loop systems, and it will be implemented here for path planning. An overview of the NMPH algorithm is presented next.

### 3.1. NMPH Algorithm for Optimal Trajectory Generation

**Figure 2:**
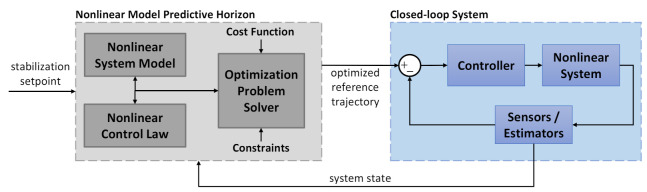
NMPH architecture. A model of the nonlinear system dynamics is used to perform the optimization process within NMPH (gray box). The resulting optimized reference trajectory is passed to the actual closed-loop system (blue box) for tracking purposes.

NMPH is an optimization-based reference trajectory generation technique for nonlinear closed-loop systems. Using a model of the nonlinear plant dynamics plus a feedback linearization control law, the NMPH creates optimal reference trajectories for a closed-loop system as shown in Figure 2. The generated trajectory is continuously updated by accounting for the current state of the system and path constraints within the optimization problem.

The purpose of the nonlinear control law within the NMPH is to reduce the nonlinearity of the system model, and consequently the non-convexity of the optimization problem. This leads to better performance in terms of reduced computational time and better convergence properties, enabling motion planning to be repeatedly computed in real time.

Consider a continuous-time nonlinear system controlled by a nonlinear control law,

(1) $$\begin{array}{cc} \dot{x} & =f\left(x,u\right) \end{array}$$

(2) $$\begin{array}{cc} \xi & =h\left(x\right) \end{array}$$

(3) $$\begin{array}{cc} u & =g(x,\xi_{{}_{ref}}) \end{array}$$

where $x\in X\subseteq R^{n_{x}}$ are the system states, $u\in U\subseteq R^{n_{u}}$ are the system inputs, and $\xi \in \Xi \subseteq R^{n_{\xi }}$ are the system outputs (here 3D position and heading, such that Ξ⊆X). The reference trajectory is denoted by $\xi_{{}_{ref}}\in \Xi$. The map f:X×U→X represents the system dynamics and g:X×Ξ→U is the control law that is used to make the system output follow the reference trajectory.

The optimization within NMPH uses Equations (1)–() plus assigned constraints to solve for the predicted system state trajectory $\tilde{x}$ (which for us includes the predicted output trajectory $\tilde{\xi }$ as a subset) and the estimated reference trajectory for the closed-loop system ${\hat{\xi }}_{ref}$, all while $\tilde{x}$ converges to a desired stabilization setpoint $x_{ss}$. The optimization calculations are summarized in Algorithm 1.

Within Algorithm 1, the optimization problem is solved over the finite time horizon $\tau \in [t_{n},t_{n}+T]$. The cost function is chosen to penalize the deviation of the predicted system state trajectory from the stabilization setpoint $x_{ss}$ , as well as the deviation of the predicted output trajectory $\tilde{\xi }$ from the estimated reference trajectory ${\hat{\xi }}_{ref}$. The weighting matrices $W_{x}$, $W_{\xi }$, and $W_{T}$ are chosen by the user to balance the effects of the optimization. The cost function consists of the stage cost function $L\left(\tilde{x}\left(\tau \right),{\hat{\xi }}_{ref}\left(\tau \right)\right)$, which represents the cost of the problem over the time horizon, and the terminal cost function $E\left(\tilde{x}\left(t_{n}+T\right)\right)$, which represents the cost of steady-state error. X⊆X, U⊆U, and Z⊆X are the state, control, and trajectory constraint sets, respectively. The inequality constraint $O_{i}\left(\tilde{x}\right)\leq 0$ is used to model *p* dynamic obstacles in the environment.

In Algorithm 1, the current system state $x\left(t_{n}\right)$ of the actual system is first measured or estimated, then a prediction of the reference trajectory ${\hat{\xi }}_{ref}$ for an admissible control is obtained by minimizing the cost function over the prediction horizon while respecting the system model, control law, and other constraints. Finally, the predicted reference trajectory is sent to the closed-loop system for tracking, and the above process is repeated at the next sampling time instant, possibly with an updated terminal setpoint $x_{ss}$.
**Algorithm 1** (NMPH algorithm with stabilizing terminal condition $x_{ss}$).**1:** Let $t_{n}$, n=0,1,2,⋯ represent successive sampling times; set n=0**2: while**$∥x_{ss}-x\left(t_{n}\right)∥\geq \delta$**do**(4)$$\begin{array}{cc} \min_{\tilde{x},{\hat{\xi }}_{ref}} & \left(\int_{t_{n}}^{t_{n}+T}L\left(\tilde{x}\left(\tau \right),{\hat{\xi }}_{ref}\left(\tau \right)\right)\;d\tau \;+\;E\left(\tilde{x}\left(t_{n}+T\right)\right)\right)=\\ \min_{\tilde{x},{\hat{\xi }}_{ref}} & (\int_{t_{n}}^{t_{n}+T}\left(∥\tilde{x}\left(\tau \right)-x_{ss}{∥}_{W_{x}}^{2}+{∥\tilde{\xi }\left(\tau \right)-{\hat{\xi }}_{ref}\left(\tau \right)∥}_{W_{\xi }}^{2}\right)\;d\tau \\ \; & +∥\tilde{x}\left(t_{n}+T\right)-x_{ss}{∥}_{W_{T}}^{2}) \end{array}$$(5)subjecttox˜(tn)=xtn,(6)x˜˙τ=fx˜τ,u˜τ,(7)u˜τ=gx˜τ,ξ^refτ,(8)x˜τ∈X,u˜τ∈U,ξ^refτ∈Z,(9)Oix˜≤0,i=1,2,…,p.         **if** $\tilde{x}\to x_{ss}$ **then** (estimated trajectory converging towards terminal setpoint)               n←n+1;         **else**               break;

In this paper, $\tilde{\xi }$ is defined as the predicted output trajectory which in our case (drone’s position and yaw angle) represents a subset of the predicted state trajectory $\tilde{x}$, while ${\hat{\xi }}_{ref}$ is the estimated reference trajectory which is computed by minimizing the cost function Equation (4) within the NMPH. ${\hat{\xi }}_{ref}$ is used as the reference trajectory for the actual closed-loop system, providing smooth trajectories which respect the dynamics of the vehicle. Please note that $\xi_{{}_{ref}}\left(n\right)$ in Equation () is taken as ${\hat{\xi }}_{ref}$, where the latter is actively updated by the NMPH algorithm in response to events such as new dynamic obstacles. Also, please note that ${\hat{\xi }}_{ref}$ and $\tilde{\xi }$ converge to each other, and both can be used as a reference trajectory for the actual closed-loop system (here the drone). Further details about the NMPH algorithm can be found in the authors’ recent work [9].

### 3.2. Feedback Linearization Control Law

This section reviews the feedback linearization (FBL) control law used within the NMPH structure to improve the prediction performance of the reference trajectory. In this work, a Multi-Input Multi-Output (MIMO) control-affine system (here a drone) is used. The FBL design for this class of systems is summarized below.

Consider a MIMO nonlinear control-affine system of the form,

(10) $$\dot{x}=f\left(x\right)+\sum_{i=1}^{n_{u}}g_{i}\left(x\right)\;u_{i}≜f\left(x\right)+G\left(x\right)u$$

where *f*,$\;g_{1},\ldots ,g_{n_{u}}$ are smooth vector fields in $R^{n_{x}}$. $G\left(x\right)$ is a $n_{x}\times n_{u}$ matrix with $\mathrm{rank}\;G\left(0\right)=n_{u}$.

The objective of FBL is to transform the nonlinear system (10) into a linear canonical form with a non-singular state feedback [11], which is defined as

(11) $$u=\beta \left(x\right)+D{\left(x\right)}^{-1}v$$

where $\beta \left(x\right)$ is a smooth function, and $\beta \left(0\right)=0$. $D{\left(x\right)}^{-1}$ is the inverse of a non-singular $n_{u}\times n_{u}$ matrix. $D\left(x\right)$ and the non-singular state feedback transformation are

(12) $$D\left(x\right)=\left[\begin{array}{ccc} \langle d\varphi_{1},ad_{f}^{r_{1}-1}g_{1}\rangle & \cdots & \langle d\varphi_{1},ad_{f}^{r_{1}-1}g_{n_{u}}\rangle \\ \vdots & \ddots & \vdots \\ \langle d\varphi_{n_{u}},ad_{f}^{r_{n_{u}}-1}g_{1}\rangle & \cdots & \langle d\varphi_{n_{u}},ad_{f}^{r_{n_{u}}-1}g_{n_{u}}\rangle \end{array}\right]$$

(13) $$v=\left[\begin{array}{c} L_{f}^{r_{1}}\varphi_{1}\\ \vdots \\ L_{f}^{r_{n_{u}}}\varphi_{n_{u}} \end{array}\right]+\left[\begin{array}{ccc} L_{g_{1}}L_{f}^{r_{1}-1}\varphi_{1} & \cdots & L_{g_{n_{u}}}L_{f}^{r_{1}-1}\varphi_{1}\\ \vdots & \ddots & \vdots \\ L_{g_{1}}L_{f}^{r_{n_{u}}-1}\varphi_{n_{u}} & \cdots & L_{g_{n_{u}}}L_{f}^{r_{n_{u}}-1}\varphi_{n_{u}} \end{array}\right]u\;,\;\;\;\;\;\;z=\left[\begin{array}{c} \varphi_{1}\\ \vdots \\ L_{f}^{r_{1}-1}\varphi_{1}\\ \vdots \\ \varphi_{n_{u}}\\ \vdots \\ L_{f}^{r_{n_{u}}-1}\varphi_{n_{u}} \end{array}\right]$$

where $\left\{\varphi_{i}\left(x\right):\langle d\varphi_{i},G_{r_{i}-2}\rangle =0,\;j\geq i,\;i=1,\ldots ,n_{u}\right\}$ are smooth functions that form the non-singular matrix $D\left(x\right)$. $r_{i}$ are the controllability indices and G is a distribution of vector fields. $L_{f}\varphi =\langle d\varphi ,f\rangle$ is the Lie derivative, $L_{f}^{r}$ means the Lie derivative is applied *r* times, and $ad_{f}^{r}g=\left[f,ad_{f}^{r-1}g\right]$ is the Lie bracket between the vector fields *f* and g. Further details about the non-singular state feedback transformation for MIMO control-affine systems can be found in Theorem 2.7.3 of reference [11].

## 4. Motion Planning in GPS-Denied Environments

### 4.1. Motion Planner Architecture

Our proposed motion planning design aims to produce optimal vehicle paths while navigating in unexplored, dynamic and GPS-denied environments. We combine a graph-based exploration technique with a Nonlinear Model Predictive Horizon-based approach based on optimization which respects the vehicle’s dynamics and supports dynamic obstacles. This integration yields feasible, optimal, and robust paths while exploring challenging environments.

**Figure 3:**
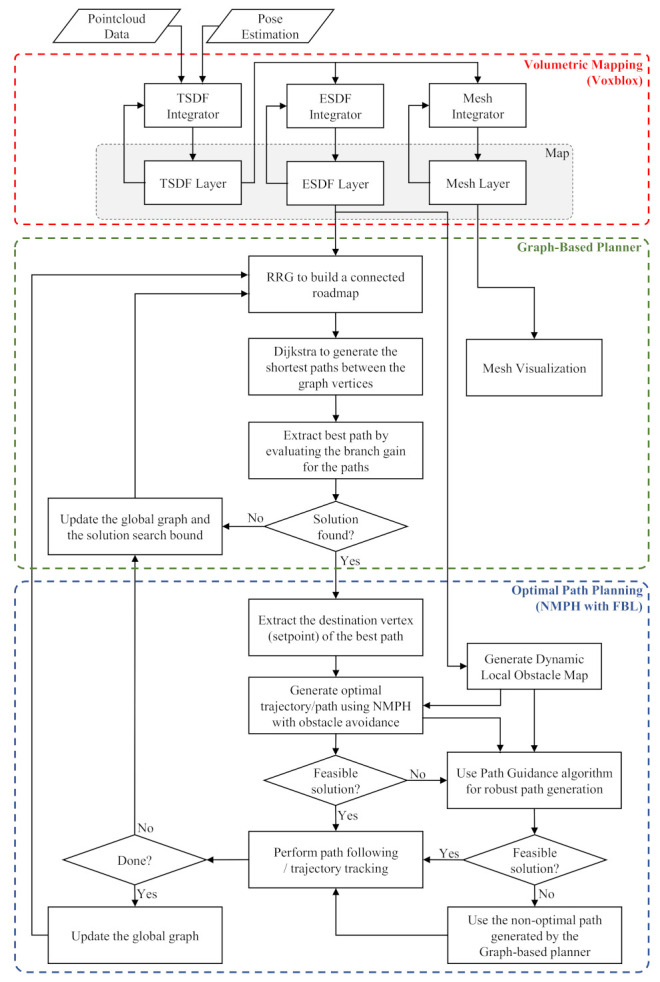
Motion Planner Architecture.

Figure 3 describes the overall architecture of our motion planner. The design is composed of three successive stages. The first stage acquires sensor data to build a physical representation of the environment which contains both static and dynamic objects in it. Volumetric mapping is used for this stage since it is computationally efficient, easy to visualize, can be incrementally constructed and reconstructed online, and provides the voxel grid structure needed for the next stage. Details about the volumetric mapping and its layers will be discussed in Section 4.2.

Section 4.3 discusses the second stage of the motion planner, which is built around a graph-based planning approach. It consists of the sampling-based RRG algorithm, which builds a connected roadmap graph, and the Dijkstra searching algorithm to extract the best path from within the graph. The main purpose of the graph-based approach is to guide the vehicle towards unexplored areas within the environment and provide terminal vertices, a.k.a. stabilization or terminal setpoints, to the local path planner.

The third stage of the motion planner uses the NMPH-FBL local path planning method. Fusing this method with the earlier stages improves the robustness of generating optimal paths and avoiding static and dynamic obstacles. The considerations involved in finding a feasible path are shown in Figure 3. Further details are provided in Section 4.4.

The reference trajectory computed by the path planner is fed to the control system of the vehicle for tracking purposes. As the drone vehicle moves, the NMPH continues to update its reference trajectory in response to feedback of the vehicle’s state and new obstacles. Once the vehicle reaches a setpoint, the motion planning process is repeated, which continues until the environment is fully explored or the mission is interrupted by the operator.

### 4.2. Volumetric Mapping

Volumetric mapping is the foundation of motion planning and navigation strategies in 3D environments. The volumetric mapping algorithm named Voxblox [32] is used in our work. In this technique, the map of the environment is represented volumetrically using the signed distance field to distinguish between known, unknown, free, and occupied spaces [33]. The grid consists of voxels with a corresponding type. Groups of occupied voxels represent surfaces of an object, walls, and so on. The main advantage of volumetric mapping is its real-time capability for incrementally representing unstructured and unexplored environments, which makes it a suitable solution for online planning and exploration. The Truncated Signed Distance Field (TSDF [34]) is one of the most efficient methods of representing volumetric maps. TSDF is an implicit surface representation that consists of a 3D voxel array. Each voxel is indexed by the distance of the ray between the sensor and the surface, and is truncated near the surface to decrease the errors that are caused by sensor noise. TSDFs are computationally efficient and can be constructed online. Also, they are capable of filtering out sensor noise and create meshes with voxel resolution for visualization purposes.

In contrast to TSDF, the Euclidean Signed Distance Field (ESDF) uses the Euclidean distance to the nearest occupied cell in labeling the voxel grid [32]. ESDFs are directly built out of existing TSDFs to make use of the distance information in determining the obstacle surface location for planning purposes. In other words, TSDF is for mapping and ESDF for planning, and the main difference between them is the way that distances are computed [35].

As presented in Figure 3, the volumetric mapping process consists of three layers. The sensor data is processed to build the TSDF layer, then the voxels are integrated into the ESDF and mesh layers as presented in [32]. The ESDF voxels and mesh blocks are updated incrementally allowing real-time map generation for planning and online visualization of the environment. To reduce the complexity of calculating the layers data, a voxel hashing approach [36] is used to store the information of each layer in a hash table, which results in O(1) complexity for adding or retrieving the data.

### 4.3. Graph-Based Path Planning

In this section, we summarize the graph-based planner presented in [5], which is used to help the vehicle navigate through unknown GPS-denied environments.

Assume that $M_{G}$ is a global 3D voxel-based map, which consists of voxels $m\in M_{G}$. The map is incrementally built by a depth sensor S plus the vehicle’s pose estimation using the volumetric mapping approach previously discussed in Section 4.2. The map is categorized into three spaces, free space $M_{G}^{f}$, occupied space $M_{G}^{o}$, and unknown space $M_{G}^{u}$. The map has a global volume $V_{G}$ and is incrementally constructed within a local map sub-space $M_{L}$ of volume $V_{L}$ centered around the current vehicle’s output (here 3D position and heading) $\xi_{0}={\left[\begin{array}{cccc} x_{0} & y_{0} & z_{0} & \psi_{0} \end{array}\right]}^{T}$.

The graph-based planner [5] performs a local search towards unknown areas of $M_{G}$. It is based on the sampling-based RRG algorithm [37] which builds a connected roadmap graph $G_{G}$ composed of collision-free vertices ν and edges *e* stored in vertex set V and edge set E, respectively. The edges are straight paths connecting the vertices using the nearest neighbor search [38]. The global graph $G_{G}$ is continuously constructed from the undirected local graph $G_{L}$ within the local space $M_{L}$. The local search within the bounded volume $V_{L}$ considers the physical size of the vehicle $V_{R}$ and bounds it within a sub-space $M_{R}$. Collision detection is performed to ensure collision-free paths $\sigma_{L}$, where $M_{R}\in M_{G}^{f}$ for all randomly generated vertices and edges.

The set of all shortest paths $\Sigma_{L}$, starting from the initial or current vertex $\xi_{0}$ to all destination vertices $\xi_{\nu }$, is found using the Dijkstra algorithm [12]. After that, the best path is evaluated by calculating the Volumetric Gain V for each vertex. The Volumetric Gain of a vertex is a measure of the unmapped volume based on the depth reading around that vertex. The weight functions related to distance and direction combined with V are used to compute the Exploration Gain $E(\sigma_{i})$ for all $\sigma_{i}\in \Sigma_{L}\;,\;i=1,\ldots ,\;n$. The vertices of these paths are $\nu_{j}^{i}\;,\;j=1,\ldots ,m_{i}$, and $\nu_{0}^{i}$ is the initial vertex along path $\sigma_{i}$. The Exploration Gain for a path is calculated as [5]

(14) $$E\left(\sigma_{i}\right)=e^{-\lambda_{S}\;S\left(\sigma_{i},\sigma_{sp}\right)}\;\sum_{j=1}^{m_{i}}V\left(\nu_{j}^{i}\right)\;e^{-\lambda_{D}\;D\left(\nu_{0}^{i},\nu_{j}^{i}\right)}$$

where $S(\sigma_{i},\sigma_{sp})$ is a distance factor between a path $\sigma_{i}$ and its corresponding straight path $\sigma_{sp}$ which has the same length along the estimated exploration direction. This factor prevents the vehicle from sudden changes in its exploration direction which might happen in branched environments within $M_{L}$. $D(\sigma_{i},\sigma_{sp})$ is the distance between $\nu_{j}^{i}$ and $\nu_{0}^{i}$ of the path $\sigma_{i}$, which penalizes longer paths for a higher exploration rate. The tunable gains $\lambda_{S}$ and $\lambda_{D}$ are positive gains.

Subsequently, the best path $\sigma_{best}$ that maximizes the Exploration Gain is selected and sent to the NMPH-FBL local motion planner to find the optimal path that the vehicle will follow. The whole procedure is repeated once the vehicle reaches the destination vertex. The detailed algorithm for building the map and planning the best path is presented in Algorithm 2.

If all vertices within $G_{L}$ are explored, the search will be expanded to the unexplored vertices of $G_{G}$. This will guide the vehicle to another location on the global map and continue the exploration mission. For the return-to-home feature, the Dijkstra algorithm is also used to find the shortest path between the vehicle’s current output $\xi_{0}$ and the homing vertex on $G_{G}$. This feature can be invoked once the exploration mission is completed, the battery level is low, or by intervention from the operator.
**Algorithm 2** Graph-based Planner.1:$\xi_{0}\leftarrow \mathrm{CurrentMeasurement}\left(\right);$2:$M_{G}\leftarrow \mathrm{VolumetricMapping}\left(S\right);$3:$V\leftarrow \left\{\xi_{0}\right\};\;\;E\leftarrow \emptyset ;\;\;G_{L}=\left(V,E\right);$4:$M_{L}\leftarrow \mathrm{LocalBoundSpace}\left(\xi_{0},M_{G}\right);$5:**for**i=1,⋯,n**do**                          ▹ RRG to build the local graph $G_{L}$6:    $\xi_{rand}\leftarrow {\mathrm{SampleFree}}_{i}\left(M_{L}\right);$7:    $\xi_{nearest}\leftarrow \mathrm{NearestVertex}\left(G_{L},\xi_{rand}\right);$8:    **if** $\mathrm{CollisionFree}(\xi_{rand},\xi_{nearest})$ **then**9:        $V\leftarrow V\cup \{\xi_{rand}\};$10:        $E\leftarrow E\cup \{\xi_{rand},\xi_{nearest}\};$11:        $N_{near}\leftarrow \mathrm{NearVertices}\left(G_{L},\xi_{rand}\right);$12:        **for** $\mathrm{each}\;\xi_{nearest}\in N_{near}$ **do**13:           **if** $\mathrm{CollisionFree}(\xi_{rand},\xi_{near})$ **then**14:               $E\leftarrow E\cup \{\xi_{rand},\xi_{near}\};$15:           endif16:        endfor17:    endif18:endfor19:$\Sigma_{L}\leftarrow \mathrm{DijkstraAlgorithm}\left(G_{L},\xi_{\nu }\right);$                    ▹ Find the shortest paths20:$\sigma_{best}\leftarrow \emptyset ;\;\;E_{best}\leftarrow 0;$21:**for**$\mathrm{each}\;\sigma \in \Sigma_{L}$**do**                               ▹ Find the best path22:    $E_{\sigma }\leftarrow \mathrm{ExplorationGain}(\sigma ,\mathrm{VolumetricGain}\left(V\right));$23:    **if** $E_{\sigma }>E_{best}$ **then**24:        $\sigma_{best}\leftarrow \sigma ;\;\;E_{best}\leftarrow E_{\sigma };$25:    endif26:endfor27:$G_{G}\leftarrow G_{G}\cup G_{L};$                              ▹ Update the global graph28:**if**ReturnHome=true**then**29:    $\sigma_{best}\leftarrow \mathrm{DijkstraAlgorithm}\left(G_{G},\xi_{home}\right);$            ▹ Find the shortest path to home30:endif

### 4.4. Nmph for Local Path Planning

The graph-based planner in Section 4.3 generates non-optimal or sub-optimal paths because the vertices are created randomly within $V_{L}$. In addition, the straight edges connecting vertices cause jerky motions for a drone following the path. Finally, the path generated by the graph-based planner does not respect the vehicle’s dynamics. The NMPH-equipped path planning approach presented in Algorithm 3 overcomes these issues by generating a path which respects the system’s dynamics and provides a smooth and optimal path which also avoids obstacles. From Figure 3, the NMPH path planning stage includes

- Dynamic Local Obstacle Mapping (c.f. Section 4.4.1), a technique which utilizes the continously updated volumetric map of the environment to generate a dynamically changing map of obstacles which are used as constraints for the optimization within the NMPH algorithm.
- Obstacle Avoidance (c.f. Section 4.4.2), an algorithm which allows the optimization problem solver to select constraints which correspond to obstacles in the path of the vehicle.
- Path Guidance (c.f. Section 4.4.3), an algorithm which enhances the robustness of path generation to infeasible situations by making use of all the vertices of the graph-based planner-generated path, not just the terminal vertex. This allows the generation of multiple consecutive and feasible paths, leading to an overall path to the terminal vertex.

**Algorithm 3** Local Optimal Path Planning using NMPH.
1:
$\sigma_{best}\leftarrow \mathrm{GraphBasedPlanner}\left(\xi_{0},M_{G}\right);$
2:
$\nu_{term}\leftarrow {\mathrm{ExtractVertex}}_{terminal}\left(\sigma_{best}\right);$
3:$M_{obs}\leftarrow \mathrm{LocalObstacleBound}\left(\xi_{0},M_{G}\right);$                            ▹ Consider certain voxels around $\xi_{0}$4:**for**i=k,⋯,n**do**                                              ▹ Remove extra voxels5:    **for** j=i−k,⋯,i **do**6:        **if** $∥m_{i}-m_{j}∥<\delta$ **then**7:           $M_{obs}\leftarrow M_{obs}\setminus m_{i};$                                    ▹ Remove $\nu_{i}$ from the obstacles map8:        endif9:    endfor10:
endfor
11:$C_{L}\leftarrow \mathrm{ObstacleConstraint}\left(\nu_{term},\xi_{0},M_{obs}\right);$                           ▹ Find the obstacles constraints12:
$\sigma_{opt}\leftarrow \mathrm{NMPH}\_\mathrm{Planning}\left(\nu_{term},\xi_{0},C_{L}\right);$
13:
**if** $\sigma_{opt}\;\mathrm{not}\;feasible$ **then**
14:    **for** i=1,⋯,n **do**                                          ▹ Path Guidance Algorithm15:        $\nu_{i}\leftarrow {\mathrm{ExtractVertex}}_{i}\left(\sigma_{best}\right);$16:        $\sigma_{opt}\leftarrow \mathrm{NMPH}\_\mathrm{Planning}\left(\nu_{i},\xi_{0},C_{L}\right);$17:    endfor18:    **if** $\sigma_{opt}\;\mathrm{not}\;feasible$ **then**19:        $\sigma_{opt}=\sigma_{best};$20:    endif21:
endif
22:$\mathrm{PathFollowing}(\sigma_{opt});$                                         ▹ Follow the optimal path


#### 4.4.1. Dynamic Local Obstacle Mapping

Transforming physical obstacles within the volumetric map to optimization constraints is a challenging task. These obstacles need to be represented by a cluster of constraints while respecting the limitations of the optimization process, specifically a limit on the number of inequality constraints that the optimization problem can handle.

In this Section, we will present a strategy that maps obstacles in the environment into a dynamically moving space around the vehicle. This facilitates representing the obstacles as inequality constraints for optimization. This mapping technique, called Dynamic Local Obstacle Mapping (DLOM), generates a continuously changing map $M_{obs}$.

**Figure 4:**
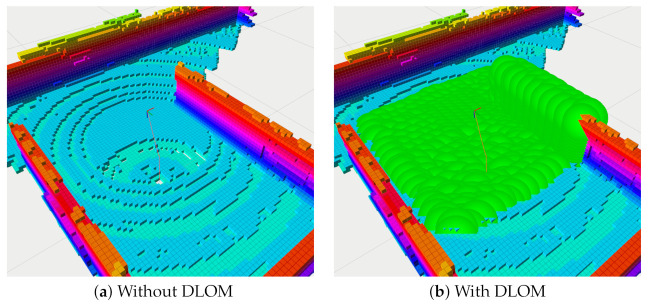
Dynamic Local Obstacle Mapping (DLOM).

Based on the occupied voxel in $M_{G}^{o}$, the DLOM strategy generates virtual spheres centered on occupied voxels within a certain space surrounding the vehicle (e.g., a box of dimensions $D_{obs}$). These virtual spheres have a radius which ensures a safe clearance between the vehicle sides and the occupied voxel. Figure 4 shows the volumetric map without and with DLOM. One advantage of using a sphere is for modeling the obstacle as a state constraint. This inequality constraint requires $r_{obs}^{i}$, the distance between the vehicle and the center of the $i^{\mathrm{th}}$ sphere, to be larger than a specific threshold $r_{thld}$ representing the radius of the virtual sphere as $\left(r_{obs}^{i}\geq r_{thld}\right)$. The solution of the optimization problem within NMPH will thus generate a path that doesn’t pass through the virtual spheres and hence avoids the obstacles in the environment.

**Figure 5:**
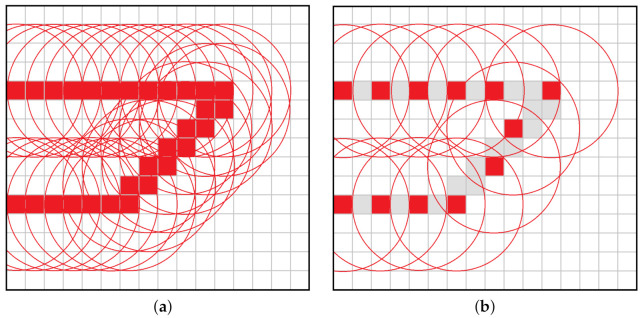
Dynamic Local Obstacle Mapping. (**a**) All voxels are used to map the obstacle’s surface. (**b**) A subset of voxels (highlighted in red) is selected to represent the obstacle’s surface, and their neighbouring voxels are excluded.

Modeling all occupied voxels in $D_{obs}$ as obstacles would result in an excessively large computational burden to continuously generate $M_{obs}$ and solve the optimization problem. Instead, any voxels inside the *i*th sphere are excluded from $M_{obs}$. Lines (3–10) of Algorithm 3 employ a simple running window strategy to remove extra voxels, and those remaining are represented as virtual spheres which provide constraints to the optimization problem. Figure 5 shows how the extra spheres are removed to reduce the computational load involved in producing the obstacles map. The exact time needed to build the dynamic obstacles map depends on the number of occupied voxels within $D_{obs}$. Experimentally, we found that the time required to build such a map on a desktop-class machine with a modern GPU (detailed specifications are given in Section 6.1) takes approximately 3ms.

#### 4.4.2. Obstacle Avoidance Algorithm

As soon as the obstacle map is created, the NMPH creates an optimal local path respecting the constraints acquired from $M_{obs}$. The optimization solver is limited in the number of inequality constraints it can handle, making it impossible to include all the mapped obstacles in $M_{obs}$ within the optimization problem. Hence, a dynamic method for selecting a specific number of constraints is described next and included in Algorithm 4.
**Algorithm 4** Obstacle Constraints.1:**function**ObstacleConstraint($\nu_{term},\xi_{0},M_{obs}$)2:    $\sigma_{opt}\leftarrow \mathrm{NMPH}\_\mathrm{Planning}\left(\nu_{term},\xi_{0}\right);$3:    k=1;4:    $C_{D}\leftarrow \emptyset ;\;C_{T}^{k}\leftarrow \emptyset ;$              ▹ Dynamic and Temporary Constraint Arrays5:    **for** j=1,⋯,N **do**                 ▹*N* is the number of the horizon points6:        **for** $i=1,\cdots ,n_{obs}$ **do**                  ▹$n_{obs}$ is the number of obstacles7:           **if** $r_{obs,\nu_{j}}^{i}<r_{thld}$ **then**8:               $C_{D}\leftarrow s_{i}^{j};$              ▹ Store *i*th obstacle position which is indexed by *j*9:               $C_{T}^{k}\leftarrow s_{i};$            ▹ Store *i*th obstacle position in the *k*th temp constraint10:               k=k+1;11:               **if** $k\;\mathrm{is}\;n_{{}_{T}}$ **then**12:                   k=1;13:               endif           14:           endif        15:        endfor    16:    endfor17:    $C_{L}=\left(C_{D},C_{T}^{k}\right);$18:**return**$C_{L}$

Our chosen solver provides a solution to the optimization problem every ∼4 ms (running on the computer described in Section 6.1), which makes it possible to solve the problem several times before sending the optimum reference path to the vehicle’s flight control system. The Obstacle Avoidance algorithm takes advantage of this by first solving the optimization problem without considering obstacle constraints, then running a collision check on the generated path to find whether it crosses any virtual spheres in $M_{obs}$. It is important to mention that the collision check is performed over the entire optimization horizon $\left[t_{n},t_{n}+T\right]$ in Algorithm 1, which is discretized into *N* points for numerical computation.

If a collision is detected at some points within the optimization horizon, a *Dynamic Constraint Array* registers the center of a sphere $\;s\in R^{3}$ that contains these collision points, and passes them to the solver as inequalities used to compute a new solution which avoids them. The *Dynamic Constraint Array* has dimensions of N×3 and can register up to *N* different inequality constraints for the next run of the optimization problem. For example, assume that a collision is detected on horizon points 3, 4 and 5, and each of the collision points are located within the 40th virtual sphere. In this case, the coordinates of the center of this sphere are registered in the *Dynamic Constraint Array* at indices 3, 4 and 5, while the rest of the array entries are kept Null. In the next iteration of the solver, a new constraint representing the cloned entries of the Dynamic Constraint Array will yield a path which avoids the region where the collisions previously occured.

To enhance the reliability of the Obstacle Avoidance algorithm while the vehicle is in motion, a specific number of Temporary Constraint Arrays (labeled by *k* in Algorithm 4) store the information from the Dynamic Constraint Array and are used in the optimization solution as well. The Temporary Constraint Arrays are static, which means that each registers only one virtual sphere over all its *N* indices.

#### 4.4.3. Robust Path Guidance Algorithm

**Figure 6:**
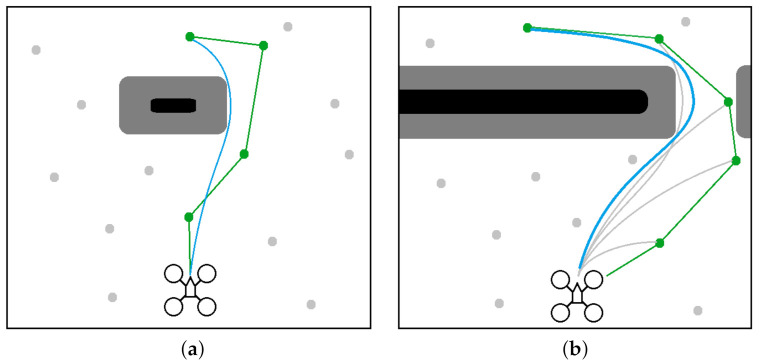
Graph-based vs NMPH path planning. (**a**) The terminal vertex of the green path (from graph-based planner) is sufficient to generate the optimum blue path by NMPH. (**b**) All the vertices of the green path are used successively to guide the solutions of NMPH to the final optimal trajectory (blue path).

The initial state of the vehicle, the nature of the environment (e.g., branched narrow passages), and the terminal condition location may all affect the feasibility of the optimization problem solution. Figure 6 depicts two different path planning scenarios. In Figure 6a, the obstacle is small and NMPH can easily find a feasible solution. In Figure 6b, the obstacles almost block the way to the destination point. In this situation, the NMPH solver risks producing infeasible solutions by getting trapped in local minima.

As mentioned in Section 4.3, the graph-based path planning yields multiple vertices, which are used by the NMPH approach to generate multiple feasible paths, ranging from the nearest to the most distal (terminal) vertex. The small obstacle depicted in Figure 6a does not cause any issues for the NMPH in generating a feasible path directly to the terminal vertex. However, Figure 6b illustrates how the NMPH algorithm uses multiple consecutive paths (gray lines) generated to the intermediate vertices of the path generated by the graph-based planner (green line) to eventually find the resulting optimal path (blue line). Lines 12–21 in Algorithm 3 demonstrate the Path Guidance algorithm that adds robustness to the NMPH approach in finding a feasible solution. Note in case the Path Guidance algorithm is unable to help NMPH find a feasible path to the terminal vertex, the system can still use the path generated by the graph-based planner.

## 5. Application of Motion Planner to A Drone

### 5.1. System Model

In this section, both a quadcopter and a hexacopter system are modeled as rigid bodies with lumped force and torque inputs at each rotor. For simplicity, drag forces, rotor gyroscopic effects, and propeller dynamics are not included in the models. The rigid-body dynamics are formulated using the Newton-Euler equations [39].

**Figure 7:**
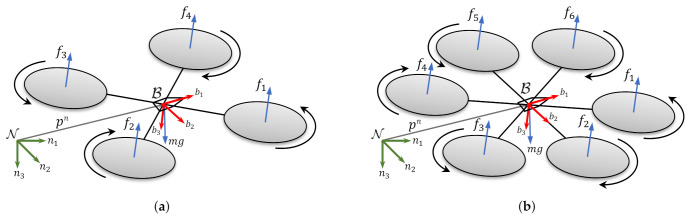
Schematics of (**a**) quadcopter and (**b**) hexacopter vehicles.

A fixed navigation frame N and a moving body-fixed frame B are the two reference frames used in this work and their basis are selected to follow the North, East, and Down (NED) aerospace convention. Schematics of the drones with their body-fixed reference frames and basis are depicted in Figure 7.

The dynamics of a rigid body moving through 3D space is represented by rigid-body kinematics and the Newton-Euler equations as shown below [39],

(15) $$\begin{array}{cc} {\dot{p}}^{n} & =v^{n}\\ m{\dot{v}}^{n} & =-\bar{u}Rn_{3}+gn_{3}\\ \dot{R} & =R\;S(\omega^{b})\\ J{\dot{\omega }}^{b} & =-S\left(\omega^{b}\right)J\omega^{b}+\tau^{b} \end{array}$$

where $p^{n}\in R^{3}$ is the vehicle’s position and $v^{n}\in R^{3}$ is its velocity, both in coordinates of the inertial navigation frame N. The mass moment of inertia matrix *J* is assumed to be diagonal as $J=\mathrm{diag}(\left[J_{1},J_{2},J_{3}\right])$, and *m* is the total mass of the drone. $\omega^{b},{\dot{\omega }}^{b}$ are the angular velocity and acceleration vectors, respectively, in coordinates of the body-fixed frame. The rotation matrix of B with respect to N is $R=R_{nb}\in \mathrm{SO}(3)$. $S\left(\cdot \right)\in R^{3\times 3}$ is a skew-symmetric matrix such that S(x)y=x×y, $x,y\in R^{3}$. The system input vector is ${\left[\bar{u},\tau^{b}\right]}^{T}$, where $\bar{u}>0$ is the net thrust from all rotors in the direction of $-b_{3}$, and $\tau^{b}={\left[\tau^{b1},\tau^{b2},\tau^{b3}\right]}^{T}$ are the torques created by the rotors about the three body frame axes.

It is important to mention that each of the vehicle configurations (quadcopter and hexacopter) transforms the rotors’ thrusts and torques to the system input vector ${\left[\bar{u},\tau^{b}\right]}^{T}$ differently. These transformations are assumed to be performed in the onboard flight controller, and consequently both configurations are represented by the same dynamics Equation (15). Hence, the proposed algorithm development is the same for both configurations.

### 5.2. Development of NMPH on a Drone Vehicle

The state and input vectors of the rigid-body dynamics presented in Equation (10) are

(16) $$\begin{array}{cc} x & ={\left[{\left(p^{n}\right)}^{T},{\left(v^{n}\right)}^{T},{\left(\eta \right)}^{T},{\left(\omega^{b}\right)}^{T}\right]}^{T}\in R^{12},\\ u & ={\left[\bar{u},{\left(\tau^{b}\right)}^{T}\right]}^{T}\in R^{4}. \end{array}$$

Using the roll-pitch-yaw (ϕ,θ,ψ) Euler angle parameterization of R∈SO(3), the nonlinear control-affine representation of Equation (10) can be expressed as

(17) $$\dot{x}=f\left(x\right)+\sum_{i=1}^{4}g_{i}\left(x\right)u_{i}≜f\left(x\right)+G\left(x\right)u$$

where

$$f\left(x\right)=\left[\begin{array}{c} x_{4}\\ x_{5}\\ x_{6}\\ 0\\ 0\\ g\\ x_{10}+s_{x_{7}}t_{x_{8}}x_{11}+c_{x_{7}}t_{x_{8}}x_{12}\\ c_{x_{7}}x_{11}-s_{x_{7}}x_{12}\\ \frac{s_{x_{7}}}{c_{x_{8}}}x_{11}+\frac{c_{x_{7}}}{c_{x_{8}}}x_{12}\\ \left(\frac{J_{2}-J_{3}}{J_{1}}\right)x_{11}x_{12}\\ \left(\frac{J_{3}-J_{1}}{J_{2}}\right)x_{10}x_{12}\\ \left(\frac{J_{1}-J_{2}}{J_{3}}\right)x_{10}x_{11} \end{array}\right],G\left(x\right)=\left[\begin{array}{cccc} 0 & 0 & 0 & 0\\ 0 & 0 & 0 & 0\\ 0 & 0 & 0 & 0\\ -\frac{1}{m}\left(c_{x_{7}}s_{x_{8}}c_{x_{9}}+s_{x_{7}}s_{x_{9}}\right) & 0 & 0 & 0\\ -\frac{1}{m}\left(c_{x_{7}}s_{x_{8}}s_{x_{9}}-s_{x_{7}}c_{x_{9}}\right) & 0 & 0 & 0\\ -\frac{1}{m}c_{x_{7}}c_{x_{8}} & 0 & 0 & 0\\ 0 & 0 & 0 & 0\\ 0 & 0 & 0 & 0\\ 0 & 0 & 0 & 0\\ 0 & \frac{1}{J_{1}} & 0 & 0\\ 0 & 0 & \frac{1}{J_{2}} & 0\\ 0 & 0 & 0 & \frac{1}{J_{3}} \end{array}\right]$$

where $s_{\left(\cdot \right)}=\sin\left(\cdot \right)$, $c_{\left(\cdot \right)}=\cos\left(\cdot \right)$, and $t_{\left(\cdot \right)}=\tan\left(\cdot \right)$.

In order to make the system in Equation (17) state feedback linearizable, the state vector is augmented with two additional states, which are the thrust $x_{13}=\bar{u}$ and its rate $x_{14}=\dot{\bar{u}}$, and the thrust is replaced by $\ddot{\bar{u}}$ in the input vector [9]. Moreover, integral states ζ are added to the system dynamics to compensate for unmodeled external disturbances which affect the control system and NMPH performances. The proposed extension of the system is presented in Equations (18) and (19).

(18) $$\begin{array}{cc} x & ={\left[{\left(p_{{}_{3\times 1}}^{n}\right)}^{T},{\left(v_{{}_{3\times 1}}^{n}\right)}^{T},{\left(\eta_{{}_{3\times 1}}^{}\right)}^{T},{\left(\omega_{{}_{3\times 1}}^{b}\right)}^{T},\bar{u},\dot{\bar{u}},{\left(\zeta_{{}_{3\times 1}}^{p^{n}}\right)}^{T},\zeta^{\psi }\right]}^{T}\in R^{18}\\ u & ={\left[\ddot{\bar{u}},{\left(\tau_{{}_{3\times 1}}^{b}\right)}^{T}\right]}^{T}\in R^{4} \end{array}$$

(19) $$\dot{x}=\bar{f}\left(x\right)+\bar{G}\left(x\right)u$$

where,

$$\bar{f}\left(x\right)=\left[\begin{array}{c} x_{4}\\ x_{5}\\ x_{6}\\ -\frac{1}{m}\left(c_{x_{7}}s_{x_{8}}c_{x_{9}}+s_{x_{7}}s_{x_{9}}\right)x_{13}\\ -\frac{1}{m}\left(c_{x_{7}}s_{x_{8}}s_{x_{9}}-s_{x_{7}}c_{x_{9}}\right)x_{13}\\ g-\frac{1}{m}c_{x_{7}}c_{x_{8}}x_{13}\\ x_{10}+s_{x_{7}}t_{x_{8}}x_{11}+c_{x_{7}}t_{x_{8}}x_{12}\\ c_{x_{7}}x_{11}-s_{x_{7}}x_{12}\\ \frac{s_{x_{7}}}{c_{x_{8}}}x_{11}+\frac{c_{x_{7}}}{c_{x_{8}}}x_{12}\\ \left(\frac{J_{2}-J_{3}}{J_{1}}\right)x_{11}x_{12}\\ \left(\frac{J_{3}-J_{1}}{J_{2}}\right)x_{10}x_{12}\\ \left(\frac{J_{1}-J_{2}}{J_{3}}\right)x_{10}x_{11}\\ x_{14}\\ 0\\ x_{1}\\ x_{2}\\ x_{3}\\ x_{9} \end{array}\right],\;\bar{G}\left(x\right)=\left[\begin{array}{c} 0_{{}_{9\times 4}}\\ \;\begin{array}{cccc} 0 & \frac{1}{J_{1}} & 0 & 0\\ 0 & 0 & \frac{1}{J_{2}} & 0\\ 0 & 0 & 0 & \frac{1}{J_{3}}\\ 0 & 0 & 0 & 0\\ 1 & 0 & 0 & 0 \end{array}\\ 0_{{}_{4\times 4}} \end{array}\right]$$

Based on the transformation presented in Section 3.2, the four smooth functions, which represent the linearizing outputs, are $\left\{\varphi_{1}\left(x\right)=x_{1},\;\varphi_{2}\left(x\right)=x_{2},\;\varphi_{3}\left(x\right)=x_{3},\;\varphi_{4}\left(x\right)=x_{9}\right\}$, and the system transformation into linear canonical form can be written as

(20) $$\begin{array}{cccc} \dot{z} & =A_{c}\;z+B_{c}\;v\;, & z & \in R^{18},\;v\in R^{4}\\ \xi & =C_{c}\;z\;, & \xi & \in R^{4} \end{array}$$

where,

$$\begin{array}{cc} z & ={\left[\varphi_{1},\cdots ,L_{f}^{r_{1}-1}\varphi_{1},\;\cdots \;,\varphi_{m},\cdots ,L_{f}^{r_{m}-1}\varphi_{m}\right]}^{T}\\ & ={\left[x_{15},x_{1},x_{4},{\dot{x}}_{4},{\ddot{x}}_{4},\;x_{16},x_{2},x_{5},{\dot{x}}_{5},{\ddot{x}}_{5},\;x_{17},x_{3},x_{6},{\dot{x}}_{6},{\ddot{x}}_{6},\;x_{18},x_{9},{\dot{x}}_{9}\right]}^{T}\\ \dot{z} & ={\left[z_{2},z_{3},z_{4},z_{5},v_{1},z_{7},z_{8},z_{9},z_{10},v_{2},z_{12},z_{13},z_{14},z_{15},v_{3},z_{17},z_{18},v_{4}\right]}^{T} \end{array}$$

The domain for a non-singular solution is $\left\{\bar{u}\neq 0,\;-\frac{\pi }{2}<\phi <\frac{\pi }{2},\;-\frac{\pi }{2}<\theta <\frac{\pi }{2}\right\}$, which is found by the determinant of matrix $D\left(x\right)$ being $\det D(x)=-\frac{{\bar{u}}^{2}\cos\left(\phi \right)}{m^{3}J_{1}J_{2}J_{3}\cos\left(\theta \right)}$. The domain limits shown above are included within the constraints of the optimization problem in NMPH ().

Finally, the feedback linearization control inputs are found using Equation (13), which are

(21) $$\begin{array}{cc} \ddot{\bar{u}}= & m\;c_{x_{9}}s_{x_{7}}v_{2}-m\;s_{x_{7}}s_{x_{9}}v_{1}-m\;c_{x_{7}}\left(s_{x_{8}}c_{x_{9}}v_{1}+s_{x_{8}}s_{x_{9}}v_{2}+c_{x_{8}}v_{3}\right)\\ \; & +x_{13}\left(x_{10}^{2}+\;x_{11}^{2}\right)\\ \tau^{b1}= & \frac{mJ_{1}}{x_{13}}s_{x_{7}}\left(s_{x_{8}}c_{x_{9}}v_{1}+s_{x_{8}}s_{x_{9}}v_{2}+c_{x_{8}}v_{3}\right)+\frac{mJ_{1}}{x_{13}}c_{x_{7}}\left(c_{x_{9}}v_{2}-s_{x_{9}}v_{1}\right)-\left(J_{2}-J_{3}\right)x_{12}x_{11}\\ \; & +\frac{J_{1}}{x_{13}}\left(x_{11}x_{12}x_{13}-\;2x_{10}x_{14}\right)\\ \tau^{b2}= & -\frac{mJ_{2}}{x_{13}}\;c_{x_{8}}\left(c_{x_{9}}v_{1}+s_{x_{9}}v_{2}\right)+\frac{mJ_{2}}{x_{13}}s_{x_{8}}v_{3}-\frac{J_{2}}{x_{13}}\left(x_{10}x_{12}x_{13}+\;2x_{11}x_{14}\right)\\ \; & +\left(J_{1}-J_{3}\right)x_{12}x_{10}\\ \tau^{b3}= & -\frac{1}{c_{x_{7}}c_{x_{8}}x_{13}}[2J_{3}\left(2x_{12}x_{11}c_{x_{7}}^{2}+s_{x_{7}}x_{11}^{2}-x_{12}^{2}c_{x_{7}}-x_{12}x_{11}\right)x_{13}s_{x_{8}}\\ \; & +\left(\left(J_{1}-J_{2}+J_{3}\right)x_{10}x_{11}x_{13}c_{x_{7}}+J_{3}s_{x_{7}}\left(ms_{x_{8}}v_{3}-2x_{10}x_{12}x_{13}-2x_{11}x_{14}\right)\right)c_{x_{8}}\\ \; & -J_{3}\left(m\;s_{x_{7}}\left(c_{x_{9}}v_{1}+s_{x_{9}}v_{2}\right)+\;x_{13}v_{4}\right)c_{x_{8}}^{2}] \end{array}$$

where the feedback inputs are selected as follows,

(22) $$v:=\left\{v_{4}=\sum_{i=16}^{18}k_{i}e_{z_{i}}\;,\;\;v_{j}=\sum_{i=5j-4}^{5j}k_{i}e_{z_{i}}\;:\;j=1,2,3\right\}$$

and the error $e_{z_{i}}$ is defined as the difference between the desired and the actual feedback state $e_{z_{i}}=z_{i}^{{}_{ref}}-z_{i}\;,\;i=1,\cdots ,n$.

**Figure 8:**
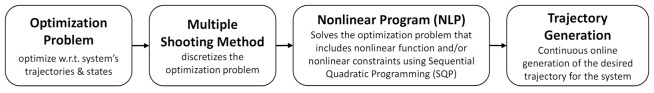
NMPH Optimization Process. The non-convex optimization problem can be solved iteratively using Sequential Quadratic Programming (SQP) [41]. In SQP, the problem is divided into a sequence of subproblems, each of which solves a quadratic objective function subject to linearized constraints [42].

As presented in Equations (15) and (21), the drone’s behavior is described by its nonlinear system dynamics and the feedback linearization control. The optimization within NMPH exploits their integration to enhance the performance of generating the reference trajectory. The continuous-time NMPH presented in Algorithm 1 is solved using a multiple shooting optimization technique. The solver used in our work, ACADO [40], discretizes the system dynamics, control law, and inequality constraints over the prediction horizon at each time instant $t_{n}$. Figure 8 shows the optimization process from the problem formulation to the trajectory generation.

The integration of the feedback linearization within the NMPH aims to reduce the non-convexity of the optimization problem. A perfect model of the system dynamics would allow the closed-loop form to be represented by a linear canonical form as shown in Equation (20), for which the feasibility and stability of the optimized solution are guaranteed and the computational power needed to solve the optimization problem is greatly reduced over the non-convex case. However, even an imperfect model still reduces the non-convexity of the optimization problem as compared to working directly with the (nonlinear) plant dynamics as in standard NMPC.

## 6. Experimental Results

In this section, simulation and a preliminary real-time hardware flight test are presented to evaluate and validate the proposed design on quadcopter and hexacopter vehicles while operating in GPS-denied environments.

The algorithms are implemented within the Robot Operating System (ROS) [43], a Linux-based system that handles communication between the individual subsystems and the vehicle. The ACADO Toolkit [40] is used for optimization. The optimization problem is programmed in a self-contained C++ environment within this toolkit, then a real-time nonlinear solver is generated to run the optimizations online. The resulting code can be compiled and run within ROS, which also handles the communication between the solver and the vehicle, either simulated or real [44]. The NMPH optimization problem (4)–() was written in C++ code using ACADO, then automatically converted into a highly efficient C code that is able to solve the optimization problem in real-time.

### 6.1. Simulation Results

In order to test the proposed approach before implementing it on a real drone, the quadcopter drone vehicle is simulated within AirSim [45]. AirSim is an open-source simulator that provides photo-realistic environments plus a physics engine to enable performing lifelike simulations.

All frameworks and the AirSim simulator are run in ROS on an Intel Core i7-10750H CPU @ 2.60–5.00 GHz equipped with the Nvidia GeForce RTX 2080 Super Max-Q GPU. The prediction horizon for NMPH was set to T=8s using a sampling time of 0.2 s, which was found satisfactory for trajectory generation. The cost function weights $W_{x}$, $W_{\xi }$, and $W_{T}$ were adjusted heuristically to ensure a balanced trajectory generation performance towards the terminal setpoint.

**Figure 9:**
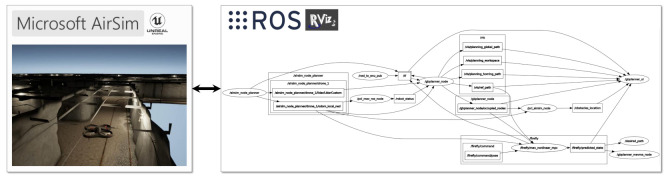
Simulation Architecture.

The drone’s measured pose and pointcloud information are obtained from the AirSim simulator and sent to the motion planner. The global graph-based and local NMPH planners generate reference trajectories for the vehicle, which are forwarded to AirSim for trajectory tracking purposes. RViz, the 3D visualization tool for ROS, is used to monitor and visualize the simulation process. Figure 9 shows the ROS network architecture of the nodes and topics employed in performing our simulation.

Different simulation results are now presented to evaluate the performance of the proposed approach on a quadcopter drone navigating autonomously through a previously unexplored, GPS-denied underground environment available within the AirSim simulator. The motion planner design illustrated in Figure 3 is implemented for this purpose. Within AirSim, the virtual quadcopter is equipped with a customized 32-channel $360^{\circ }$ scanning Lidar sensor with a $45^{\circ }$ vertical field of view, 10 Hz rotation rate, and 50 m range. The pointcloud data plus the vehicle pose are acquired and used to build a volumetric map of the environment and locate the vehicle within it.

As discussed in Section 4.3, the graph-based planning algorithm guides the vehicle towards unexplored areas within the map and provides vertices as terminal setpoints $x_{ss}$ for the NMPH local path planner. The design’s robustness is increased by implementing the Obstacle Avoidance and Path Guidance algorithms proposed in Section 4.4.2 and Section 4.4.3, respectively. Finally, the generated reference path from the motion planner is sent to the AirSim quadcopter for tracking. The NMPH continues updating the path during the vehicle’s movement toward a setpoint. This allows to avoid dynamic obstacles and improves the tracking performance. Once the vehicle reaches a setpoint, the planning process is repeated until the environment is fully explored or the mission is interrupted by the operator.

**Figure 10:**
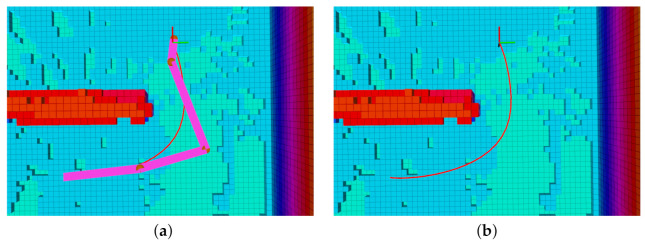
Motion Planner. (**a**) Path planning using graph-based approach (pink) and NMPH algorithm (red). (**b**) Optimal path using NMPH algorithm.

Figure 10 shows the paths generated by the graph-based and the NMPH path planners. The NMPH is seen to provide a smooth and optimal path as compared to the sharp-corner path generated by the graph-based planner. Moreover, the NMPH keeps updating the path dynamically from the start to the terminal point at a rate of up to 100 Hz, while the graph-based planner generates only one path between the two points. To reduce computational load, the NMPH algorithm rate is set to 5 Hz, which was found to be suitable in generating continuous and smooth paths in the environment. This rate of path regeneration also provides good path following performance in the presence of static and dynamic obstacles.

**Figure 11:**
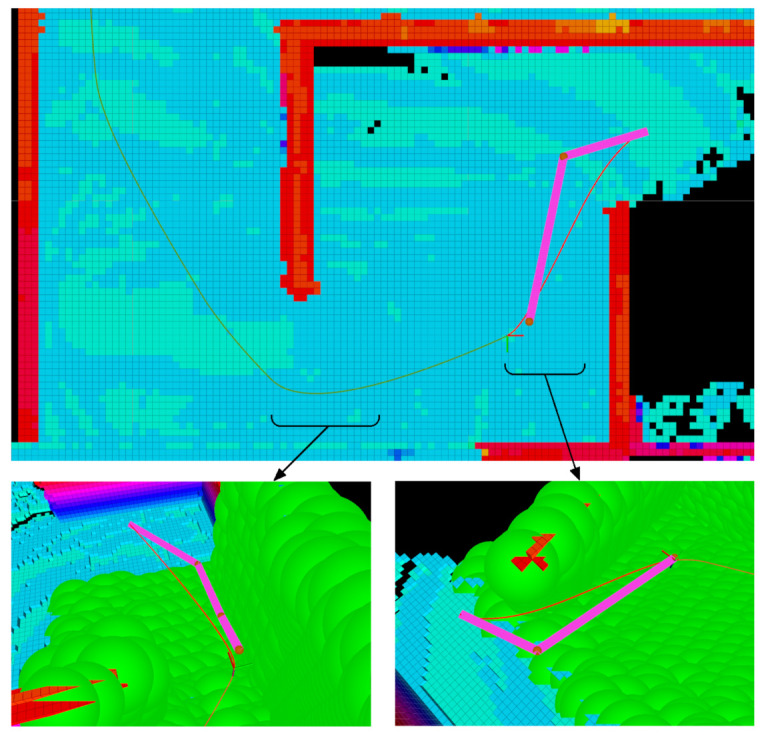
Illustration of trajectory generation and tracking. The green path is the trajectory of the vehicle, and the red path is the future reference path.

A portion of the overall tracked trajectory between multiple vertices using the NMPH algorithm can be seen in Figure 11. Respecting the system dynamics provides smooth flight paths and thus reduces power consumption caused by abrupt changes in the trajectory.

**Figure 12:**
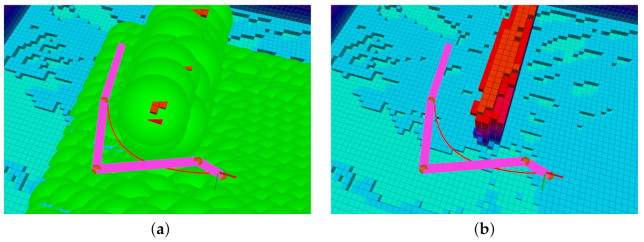
Dynamic Local Obstacle Mapping and Avoidance. In (**a**) the DLOM is made visible while in (**b**) it is hidden.

The DLOM generates a continuously changing obstacle map modeled by virtual spheres as depicted in Figure 12. As discussed in Section 4.4.1 and Section 4.4.2, mapped obstacles are represented by (continuously updated) inequality constraints within the optimization problem. The Obstacle Avoidance Algorithm helps in creating and updating a path that avoids the obstacles as shown in Figure 12.

**Figure 13:**
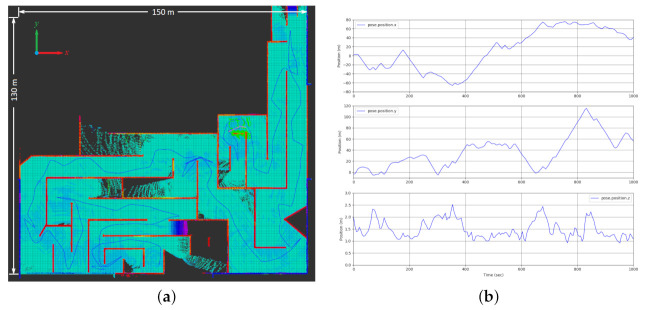
Autonomous navigation and exploration in GPS-denied environment. The vehicle travelled a total distance of 774.5m in about 1035s. (**a**) Overhead visualization of exploration path through environment. (**b**) Plot of vehicle positions (x,y,z) versus time.

**Figure 14:**
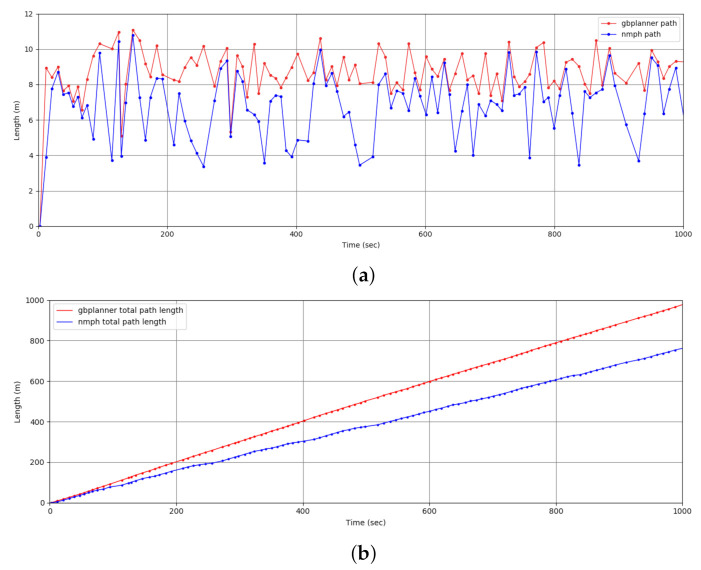
Comparison of path lengths between graph-based planner and our proposed NMPH path planner. (**a**) Path length between stabilization points. (**b**) Total length of generated paths.

In the next simulation test, the quadcopter autonomously navigates an unexplored GPS-denied environment. Figure 13 shows the exploration mission performed by the quadcopter. The drone travels a total distance of 774.5m while following smooth trajectories generated by our proposed algorithm. Meanwhile, the graph-based planner generated paths with a total length of 993.1m for the same exploration mission. Table 1 and Figure 14 offer a mission performance comparison between using the graph-based planner solo versus using the graph-based planner integrated with our NMPH approach in terms of exploration time, average computation time of the generated paths, path lengths between terminal vertices, and average and total length of the generated paths. This comparison shows the impact of using the NMPH algorithm for reducing power consumption, total mission time, and unwanted abrupt motions while following the generated reference paths.

**Figure 15:**
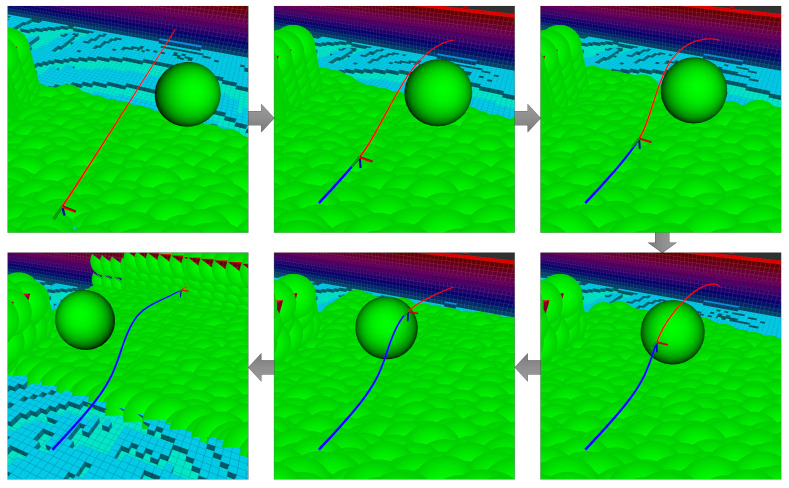
Obstacle avoidance for a moving object. The object (sphere) is moving to the left. The NMPH regenerates the red path continuously to avoid the object. The blue curve represents the flight trajectory of the drone.

In the final simulation test, obstacle avoidance for a moving object is tested while the drone navigates through the environment. This is shown in Figure 15, where the continuous regeneration of the path by the NMPH algorithm enables the drone to safely navigate to the stabilization point.

### 6.2. Preliminary Real-Time Flight Test Results

For real-time hardware testing, a FlameWheel F550 hexacopter (DJI Technology, Shenzhen, China) was used. The vehicle is equipped with a Pixhawk 2.1 autopilot control board running the PX4 flight control software [46], and an onboard Jetson TX2 (NVidia, Santa Clara, CA, USA) computer running ROS. The communication between ROS and PX4 is established through MAVLink. A RealSense T265 stereo camera and a RealSense L515 LiDAR camera (Intel, Santa Clara, CA, USA) are mounted on the hexacopter to provide pose and RGB-D pointcloud data, respectively.

**Figure 16:**
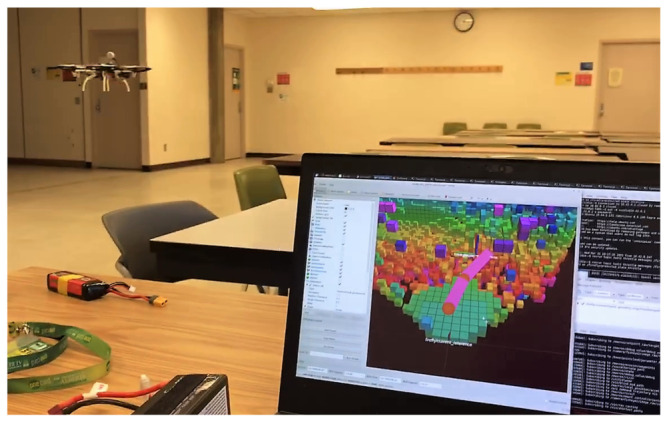
Preliminary flight test.

The preliminary flight test evaluates the path generation performance of NMPH running onboard a real drone, whose Jetson TX2 has lower computational power than the computer used in simulation. Also, local trajectory tracking and the functionality of the motion planner are tested in this experiment, as shown in Figure 16. Note that hardware flights in large-scale areas will be performed and evaluated in future work.

**Figure 17:**
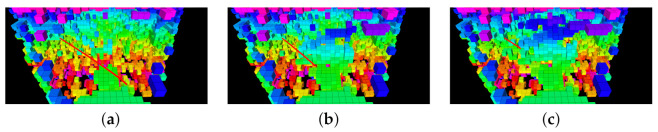
Hardware flight testing: successive captures of screen (**a**–**c**) displaying sensed environment (blocks) and planned path (red line) of drone.

In the current setup, the optimization solver within NMPH was able to provide continuous regeneration of the reference trajectories at rates approaching 70 Hz. This rate was reduced to 5 Hz to minimize the computational load on the onboard system. The graph-based motion planner was also tested by generating terminal points for NMPH. The latter sent the predicted reference trajectories to the onboard PX4, and the hexacopter was able to follow them. Figure 17 shows the hexacopter following a continuously regenerated reference path to a terminal vertex.

## 7. Conclusions

This paper presented a methodological motion planning approach for drone exploration in GPS-denied environments, which integrates our recently proposed NMPH path planning approach with a graph-based planner. The NMPH formulation employs the nonlinear system dynamics model with feedback linearization control inside an online optimization-based process to generate feasible, optimal and smooth reference trajectories for the vehicle. The performance of the overall motion planner is increased by introducing methods for robust path generation and dynamic obstacle mapping and avoidance.

The developed motion planner was evaluated through a series of simulation flights as well as a real-time hardware flight test to validate the performance of the proposed design on quadcopter and hexacopter drones navigating the environment. The results show the ability our algorithm to improve motion planning performance over conventional techniques and generate smooth and safe flight trajectories in a computationally efficient way.

Future work will include testing the proposed motion planning methodology inside large-scale GPS-denied environments such as subterranean mines.

## Figures and Tables

**Table 1:** Comparison between Graph-based and Graph-based-plus-NMPH approaches to path planning.

	Total Length of the Generated Paths	Average Path Length (between Terminal Points)	Average Path Computation Time	Exploration Time	Continuous Path Generation
Graph-based	993.1m	8.79m	733ms	1327s	No
Graph-based- plus-NMPH	774.5m	6.86m	4.34ms	1035s	Yes

## Data Availability

Data sharing not applicable.
